# Sudden life-threatening laryngeal edema in pregnancy: a case report

**DOI:** 10.1186/s13256-023-03880-7

**Published:** 2023-04-20

**Authors:** Vincent Chrisnata, Adhrie Sugiarto, Erniody Erniody, Aldy Sethiono

**Affiliations:** 1 Department of Anesthesiology and Intensive Care, Faculty of Medicine, Universitas Indonesia, Ciptomangunkusumo Hospital, Jakarta, Indonesia; 2 Department of Anesthesiology and Intensive Care, Husada Hospital, Jakarta, Indonesia

**Keywords:** Laryngeal edema, Preeclampsia, Intensive care unit, Cesarean section, Case report

## Abstract

**Background:**

Severe laryngeal edema during pregnancy is uncommon but can be encountered, particularly in patients with preeclampsia accompanied by other comorbidities. Careful consideration must be given to balance the urgency of securing the airway with the safety of the fetus and the patient’s long-term health consequences.

**Case presentation:**

A 37-year-old Indonesian woman was brought to the emergency department at 36 weeks gestation due to severe dyspnea. Her condition worsened a few hours later during intensive care unit admission, with tachypnea, decreased oxygen saturation, and inability to communicate, necessitating intubation. Due to the edematous larynx, we could only use 6.0-sized endotracheal tube. The use of a small-sized endotracheal tube was expected to be short-lived, so she was considered for tracheostomy. Nevertheless, we decided to perform a cesarean section first after lung maturation because it would be safer for the fetus, and laryngeal edema usually improves after delivery. Cesarean section was performed under spinal anesthesia for the safety of the fetus, and 48 hours after delivery, she underwent a leakage test with a positive result, so extubation was performed. Stridor was no longer audible, breathing pattern was within normal limits, and vital signs were stable. The patient and her baby both recovered well with no long-term health consequences.

**Conclusion:**

This case demonstrates that unexpected life-threatening laryngeal edema can occur during pregnancy, in which upper respiratory tract infections may trigger it. The decision between conservative and aggressive immediate airway management should be made with careful consideration of securing the patient’s airway, the safety of the fetus, and the patient’s long-term health consequences.

## Background

During pregnancy, the body undergoes many physiological changes, including changes to the larynx. Manifestations can range from minor voice changes to life-threatening airway obstruction [1]. Severe laryngeal edema during pregnancy is uncommon but can be encountered, particularly in patients with preeclampsia accompanied by other comorbidities [2]. Tracheostomies must be carefully considered to balance the urgency of securing the airway with the safety of the fetus and the patient’s long-term health consequences. We present our experience treating a woman with preeclampsia with a history of thyroidectomy, recurrent upper respiratory tract infection, and gastroesophageal reflux disease (GERD) who developed sudden, life-threatening laryngeal edema.

## Case presentation

A 37-year-old Indonesian woman was brought to the emergency department (ED) at 36 weeks gestation in her second pregnancy with shortness of breath that worsened 1 day before admission. She had a fever and bloody phlegm cough within the last 2 days, accompanied by painful swallowing and hoarseness for 2 months. She also had worsening gastroesophageal reflux disease (GERD) without medication, as well as a history of thyroidectomy due to malignancy 2 years ago.

Upon arrival, she had a respiratory rate of 26–30 breaths per minute, SpO_2_ of 98% on nasal oxygen supplementation, pulse rate of 110–120 beats per minute, and blood pressure of 172/92 mmHg. The pregnancy ultrasound showed a single live intrauterine fetus with a normal fetal heart rate [estimated fetal weight (EFW) 2184 g], corresponding to 36 weeks of gestation, and no cervical dilation. Laboratory tests revealed leukocytosis of 17,000/µL and proteinuria. Other tests such as albumin, electrolytes, and coagulation profile were within normal range. A preliminary diagnosis of pneumonia or heart failure was made in the ED. The patient was initially treated with ceftizoxime 1 g injection t.i.d. and furosemide 20 mg injection, and was planned to be admitted to the intensive care unit (ICU). However, later the chest X-ray showed neither signs of infiltrates nor pulmonary edema, and echocardiography showed normal left and right heart function.

We suspected laryngeal edema in the ICU due to the patient’s preeclamptic condition and stridor, which was initially treated with an empirical methylprednisolone injection of 125 mg t.i.d. and nebulized epinephrine. Pantoprazole 40 mg injection b.i.d. was also administered to overcome GERD.

A total of 16 hours after admission to the ICU, her condition deteriorated, with worsened tachypnea, decreased oxygen saturation, and an inability to talk, so we decided to perform intubation. Mask ventilation was used for preoxygenation, and no difficulty was encountered. Intubation was facilitated by administering propofol (1.5 mg/kg BW) and fentanyl (1 μg/kg BW). We could only use 6.0-sized endotracheal tube (ETT) because of the edematous larynx. We stopped giving sedation after the patient could tolerate the use of ETT. Pressure support ventilator mode and spontaneous breathing with T-tube were used, taking turns.

**Fig. 1:**
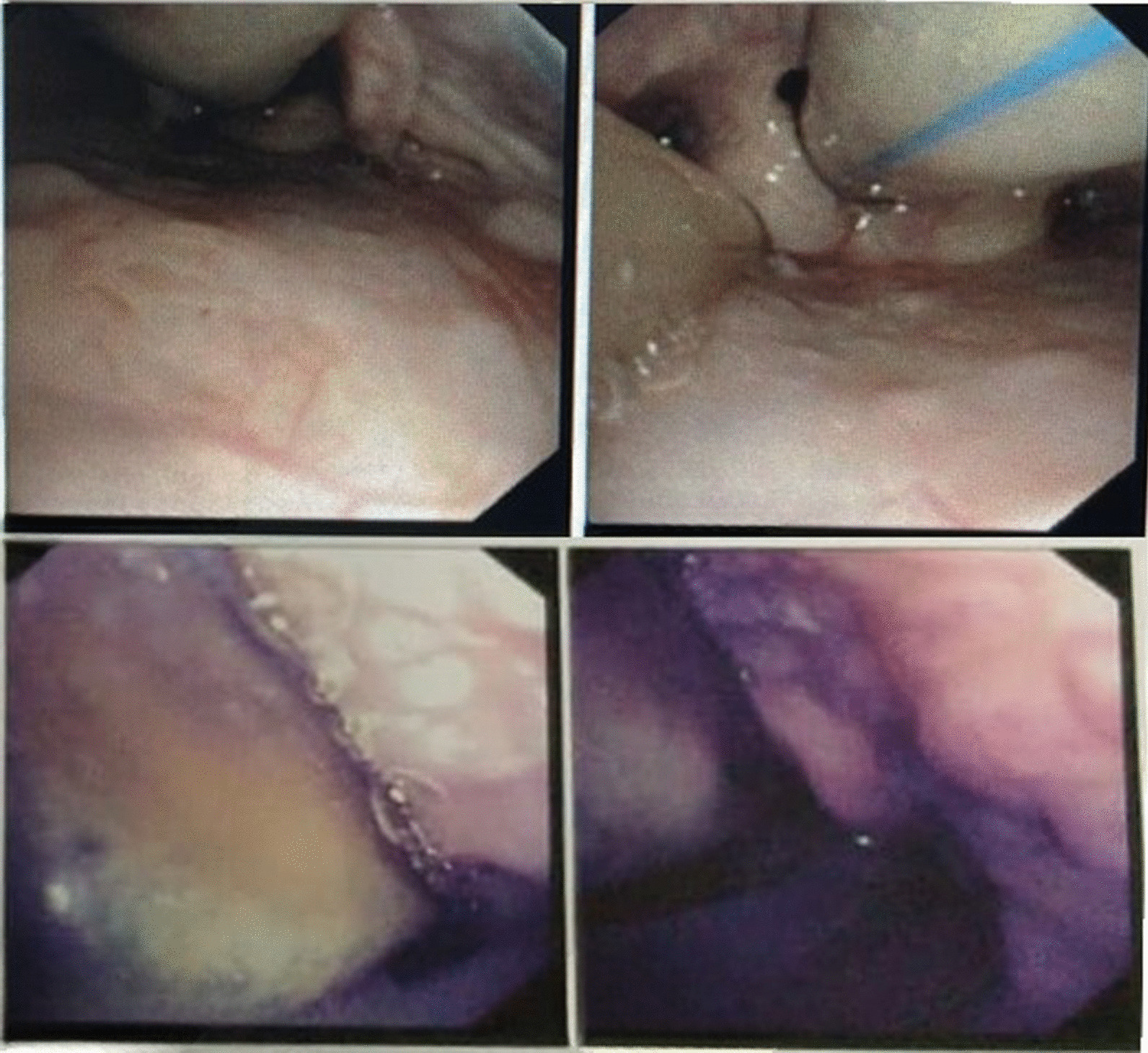
Bronchoscopy findings showing laryngeal edema with multiple white plaques

The following days, a fiberoptic bronchoscopy revealed uneven granular vocal cords with multiple white plaques and a swollen red appearance due to laryngeal edema (Fig. 1). Due to a suspicion of fungal infection based on bronchoscopy findings, methylprednisolone was stopped and additional treatment of fluconazole 800 mg, then 400 mg q.d., was given.

The use of a small-sized ETT was expected to be short-lived, so she was considered for tracheostomy. Nevertheless, we decided to perform a cesarean section first after lung maturation because it would be safer for the fetus, and laryngeal edema usually improves after delivery.

**Fig. 2:**
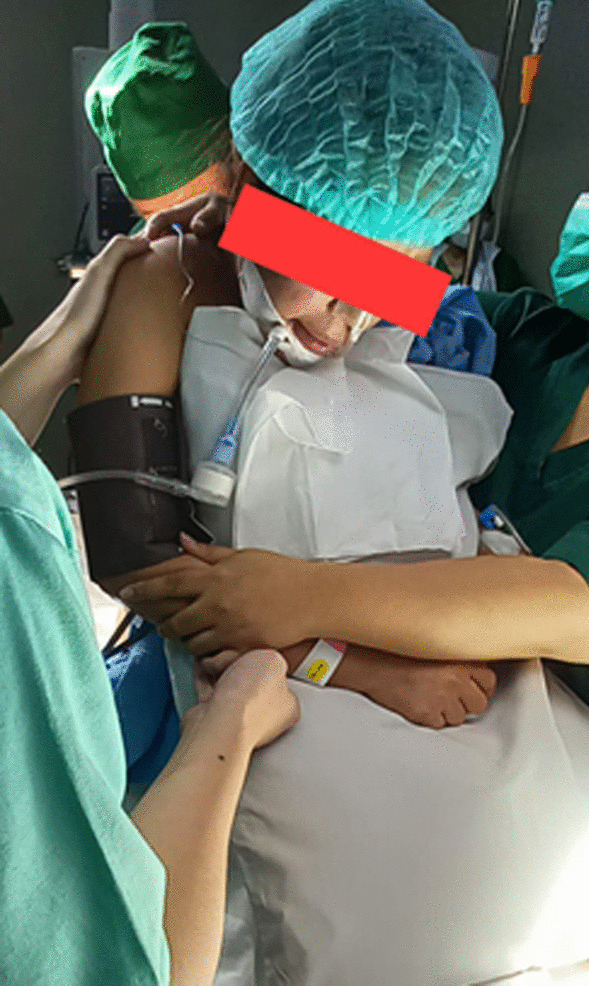
Patient undergoing spinal anesthesia while still on ETT

Despite the fact that the patient was still using ETT, she was utterly cooperative, so spinal anesthesia was given instead of general anesthesia (Fig. 2). Hyperbaric bupivacaine 10 mg with fentanyl 25 μg was administered from the L4/L5 interspace. There were no intraoperative problems. She was readmitted to the ICU after surgery with no significant complaints, and 48 hours after delivery, her clinical and vital signs were stable.

Extubation was performed after a positive result from the leakage test. Stridor was no longer audible, breathing pattern was within normal limits, and vital signs were stable, although the patient still could not talk. She was transferred to the ward for postpartum care. A laryngeal lavage fluid sample was taken during the bronchoscopy for culture, and a week later, the result came back with *Enterobacter aerogenes*.

After 2 days of treatment in the ward, the patient was discharged from the hospital. She returned to our hospital 2 weeks later for a post-discharge follow-up, and her condition improved. The baby was also in good health. Table 1 presents a timeline of the case history.

**Table 1:** Timeline of the case report

Time	Relevant past medical history and interventions
Years ago	GERD without any medications
–2 years	Enlargement of the thyroid gland, underwent thyroidectomy Biopsy result: thyroid malignancy Sometimes had hoarseness and recurrent common cold since thyroidectomy Routine medications: gefitinib 250 mg q.d. and levothyroxine sodium 100 mcg q.d., but stopped on her own initiative during pregnancy

Time	Summaries of initial and follow-up visits	Diagnostic testing	Interventions
Admission	Emergency department: History: → 36 weeks gestation → Shortness of breath since 1 day ago → Fever and bloody phlegm cough since 2 days ago → Painful swallowing, hoarseness, worsened GERD since 2 months ago Diagnosis: → Preeclampsia → Dyspnea due to suspect pneumonia or heart failure	Physical examinations: → RR 26–30 breaths/minute → HR 110–120 beats/minute → BP 172/92 mmHg → SpO_2_ 98% 6 LPM, nasal → Temperature 37.5 °C → Stridor (+) → Fetal HR 144 beats/minute → No cervical dilation Laboratory results: → Leukocytosis 17,000/µL → Protein urine (+)	Ceftizoxim 1 g intravenous t.i.d Furosemide 20 mg intravenous Dexamethasone 6 mg intravenous every 12 hours for lung maturation
+4 hours	ICU: Suspect laryngeal edema	USG: → Single live intrauterine fetus → EFW 2184 g → Gestational age 36 weeks → Sufficient amniotic fluid	Methylprednisolone 125 mg intravenous t.i.d N-acetylcysteine 400 mg t.i.d Nebulized epinephrine t.i.d
+20 hours	ICU: Worsened dyspnea Could not talk	RR 34 breaths/minute HR 145 beats/minute BP 146/82 mmHg SpO_2_ 99% 10 LPM, NRM	Intubation with 6.0-sized ETT Propofol (1.5 mg/kg BW) and Fentanyl (1 μg/kg BW)
	ICU: Post-intubation Spontaneous breathing (+) Tolerated ETT well		Pressure support ventilator mode and spontaneous breathing with T-tube were used, taking turns
+1 day	ICU: Monitoring	Echocardiography: Normal right and left heart function Bronchoscopy: Uneven granular vocal cords with multiple white plaques and swollen red appearance → laryngeal edema, suspect a fungal infection	– Fluconazole 800 mg then 400 mg q.d Pantoprazole 40 mg intravenous b.i.d Tracheostomy plan Cesarean section plan
+2 days	Operating room: The patient fully cooperated, no intraoperative problems		Cesarean section with spinal anesthesia (hyperbaric bupivacaine 10 mg and Fentanyl 25 μg) Oxygen via T-piece
	Postoperative		Monitoring in ICU
+4 days	ICU: Shortness of breath improved	Leakage test (+)	Extubation
+5 days	ICU: Post-extubation, no stridor		Transferred to the ward
+7 days	Patient was discharged	Laryngeal lavage fluid culture: *Enterobacter aerogenes*	
+2 weeks	During the post-discharge follow-up, both the patient and the baby were in good health		

*GERD* gastroesophageal reflux disease, *mg* milligram, *mcg* microgram, *RR* respiratory rate, *HR* heart rate, *BP* blood pressure, *LPM* liters per minute, *NRM* non-rebreather mask, *ICU* intensive care unit, *USG* ultrasonography, *EFW* estimated fetal weight, *ETT* endotracheal tube, *t.i.d.* ter in die (three times a day), *q.d.* qua que die (once a day), *b.i.d.* bis in die (twice a day)

## Discussion and conclusion

A 36-week pregnant woman experienced high blood pressure and proteinuria; hence, the diagnosis of preeclampsia was made. Dyspnea with fever and cough was initially thought to be caused by pneumonia or heart failure; however, chest X-ray and echocardiography results were normal. In the ICU, dyspnea with stridor was suspected to be caused by laryngeal edema, which was proven during intubation and supported by bronchoscopy findings.

During pregnancy, physiological changes to the body also involve the airway system. The surge of estrogen and progesterone causes an increase in permeability and changes the microvasculature of the lamina propria just below the laryngeal mucosa. Laryngeal mucosal swelling usually disappears after delivery, but sometimes it may persist. Certain conditions may increase the risk of laryngeal edema, the most frequent being preeclampsia, that is, generalized edema throughout the body due to fluid retention. Nonetheless, laryngeal edema caused solely by preeclampsia is quite rare. Other precipitating factors are laryngitis and a history of thyroidectomy [2–4].

In our case, there is a possibility that multiple factors cause laryngeal edema. The patient has had a history of GERD for a long time without medications, which worsened over the last 2 months. GERD is seen in 30–50% of pregnant women and worsens in the third trimester due to increased abdominal pressure, decreased gastric emptying, and decreased lower esophageal sphincter tone under the influence of progesterone [5].

Such reflux of gastroduodenal contents might directly damage the laryngeal mucosa [6]. Swain *et al*. found that 46.29% of pregnant women presented with LPR symptoms, which included hoarseness and odynophagia, similar to the patient’s complaints [1]. Treatment of this condition during pregnancy consists of lifestyle modifications and dietary changes; however, more severe symptoms might require pharmacological therapy. We used lansoprazole, a proton pump inhibitor (PPI), for this patient. This drug class is the most effective inhibitor of gastric acid secretion currently available [5].

Previously, the patient had a history of thyroidectomy; after that, she often complained about hoarseness and the common cold. There is a possibility of recurrent laryngeal nerve injury, which may cause paralysis of the vocal cords and increase the risk of infection in the upper respiratory tract, including the larynx [4, 7]. Viruses are the most common cause of acute laryngitis, most often rhinoviruses, adenoviruses, influenza, and parainfluenza. Bacterial infection is another etiology that is difficult to distinguish from viral infection. A fungus infection is also common yet underdiagnosed, which appears as whitish speckling of the supraglottis or glottis, diffuse laryngeal erythema, and edema, as in the findings of this patient [8, 9]. However, the culture results showed a bacterial, not fungal, infection.

On the basis of the findings of fever and bloody phlegm cough followed by shortness of breath and the laryngeal lavage fluid culture results, we suggested bacterial laryngitis as the precipitating factor of sudden life-threatening laryngeal edema in this patient, who already had several predisposing factors, that is, preeclampsia, GERD, reflux laryngitis, and possible recurrent laryngeal nerve injury. This patient had already been experiencing recurrent upper respiratory tract infections during her pregnancy, but laryngeal edema occurred at 36 weeks gestation. It was possibly due to hormonal factors that resulted in swelling of the airway mucosa.

The management of laryngeal edema varies depending on its severity and causes. Patients with a compromised airway should receive immediate treatment. Methylprednisolone and epinephrine inhalation have been known to be used to treat post-extubation laryngeal edema [10]. Antibiotics are still debatable and only indicated in certain conditions suspected of being caused by an infection [9]. Many cases of laryngeal edema during pregnancy necessitated a tracheostomy and the termination of the pregnancy under general anesthesia [2]. However, we decided to postpone the tracheostomy in this case because the patient could breathe comfortably with the ETT in place. Hence, considering the risk of tracheostomy and the safety of the fetus, we performed the cesarean section after ensuring the fetus’s lung maturation. Failure to perform a tracheostomy may cause airway obstruction and impair the oxygenation of both mother and fetus.

Aside from the airway issues, the patient was relatively stable and cooperative, allowing us to perform a cesarean section under spinal anesthesia, which was safer, despite the patient’s continued use of the ETT. With the pregnancy’s termination and the placenta’s removal at delivery, hormone levels such as estrogen and progesterone fall dramatically [11]. The reduction in these hormone levels may aid in the reduction of airway edema. Furthermore, with appropriate preeclampsia management and the administration of antibiotics, antiinflammatories, and a proton pump inhibitor, the patient’s condition improved enough to allow her to be extubated.

To the best of our knowledge, this is the first case report of emergency laryngeal edema in pregnancy managed conservatively without a tracheostomy and with a cesarean section performed under spinal anesthesia. The patient’s condition gradually improved after delivery. The limitation in our case was that we did not reevaluate the patient using fiberoptic bronchoscopy before extubating, instead only performing a leak test.

The lesson learned from this case is that sudden, life-threatening laryngeal edema can occur during pregnancy, and upper respiratory tract infections may trigger it. Laryngeal manifestations resolve completely after delivery and appropriate treatment for the infection. Therefore, the decision between conservative and aggressive immediate airway management should be made with careful consideration of securing the patent airway, the safety of the fetus, and the patient’s long-term health consequences.

## Data Availability

All relevant data are within the manuscript. More clinical information is available from the corresponding authors upon reasonable request.

## Abbreviations

ICU: Intensive care unit
ETT: Endotracheal tube
ED: Emergency department
KgBW: Kilogram body weight
GERD: Gastroesophageal reflux disease
EFW: Estimated fetal weight
USG: Ultrasonography
LPR: Laryngopharyngeal reflux
RR: Respiratory rate
HR: Heart rate
BP: Blood pressure
LPM: Liters per minute
NRM: Non-rebreathing mask
t.i.d.: Ter in die (three times a day)
q.d.: Quaque die (once a day)
b.i.d.: Bis in die (twice a day)
iv: Intravenous
